# Serum Complement C3 and C4 and COVID-19 Severity and Mortality: A Systematic Review and Meta-Analysis With Meta-Regression

**DOI:** 10.3389/fimmu.2021.696085

**Published:** 2021-06-07

**Authors:** Angelo Zinellu, Arduino A. Mangoni

**Affiliations:** ^1^ Department of Biomedical Sciences, University of Sassari, Sassari, Italy; ^2^ Discipline of Clinical Pharmacology, College of Medicine and Public Health, Flinders University, Adelaide, SA, Australia; ^3^ Department of Clinical Pharmacology, Flinders Medical Centre, Southern Adelaide Local Health Network, Adelaide, SA, Australia

**Keywords:** complement system, C3, C4, COVID-19 severity, mortality

## Abstract

Activation of the complement system has been observed in coronavirus disease 19 (COVID-19). We conducted a systematic review and meta-analysis with meta-regression to investigate possible differences in the serum concentrations of two routinely measured complement components, C3 and C4, in COVID-19 patients with different severity and survival status. We searched PubMed, Web of Science and Scopus, between January 2020 and February 2021, for studies reporting serum complement C3 and C4, measures of COVID-19 severity, and survival. Eligibility criteria were a) reporting continuous data on serum C3 and C4 concentrations in COVID-19 patients, -b) investigating COVID-19 patients with different disease severity and/or survival status, c) adult patients, d) English language, e) ≥10 patients, and f) full-text available. Using a random-effects model, standardized mean differences (SMD) with 95% confidence intervals (CIs) were calculated to evaluate differences in serum C3 and C4 concentrations between COVID-19 patients with low vs. high severity or survivor vs. non-survivor status. Risk of bias was assessed using the Newcastle-Ottawa scale whereas publication bias was assessed with the Begg’s and Egger’s tests. Certainty of evidence was assessed using GRADE. Nineteen studies in 3,764 COVID-19 patients were included in the meta-analysis. Both C3 and C4 concentrations were significantly lower in patients with high disease severity or non-survivor status than patients with low severity or survivor status (C3 SMD=-0.40, 95% CI -0.60 to -0.21, p<0.001; C4 SMD=-0.29, 95% CI -0.49 to -0.09, p=0.005; moderate certainty of evidence). Extreme between-study heterogeneity was observed (C3, I^2^ = 82.1%; C4, I^2^ = 84.4%). Sensitivity analysis, performed by sequentially removing each study and re-assessing the pooled estimates, showed that the magnitude and direction of the effect size was not modified. There was no publication bias. In meta-regression, the SMD of C3 was significantly associated with white blood cell count, C-reactive protein (CRP), and pro-thrombin time, whereas the SMD of C4 was significantly associated with CRP, pro-thrombin time, D-dimer, and albumin. In conclusion, lower concentrations of C3 and C4, indicating complement activation, were significantly associated with higher COVID-19 severity and mortality. C3 and C4 might be useful to predict adverse clinical consequences in these patients.

**Systematic Review Registration:** PROSPERO, Registration number: CRD42021239634.

## Introduction

The complement system exerts several protective effects against infectious agents following activation during innate, through the alternative and lectin pathways, and acquired, through the classical pathway, immunity (1). The activation of the classical, lectin, and alternative pathways ultimately leads to the cleavage of the central component 3, C3, by convertases. This, in turn, initiates a sequence of events that include phagocytosis, leucocyte attraction and activation, mast cell and basophil degranulation with the release of several mediators of inflammation, activation of the inflammasome complex and specific cytokines, and B lymphocyte activation with the consequent secretion of specific antibodies (2). In the setting of viral infections, additional effects mediated by the activation of the complement system include virus aggregation-mediated neutralization, phagocytosis, and lysis of viruses and virus-infected cells (3). While these processes suggest an overall beneficial effect against viruses, complement activation might also increase the risk of adverse clinical outcomes, in virtue of the sustained release of pro-inflammatory mediators with additional toxic effects at the cellular and tissue level (4).

The potential opposite nature of the effects mediated by the complement system has been investigated during the last three pandemics, caused by the viral agents, severe acute respiratory syndrome coronavirus, Middle East respiratory syndrome coronavirus, and severe acute respiratory syndrome coronavirus 2 (SARS-CoV-2), respectively. In particular, the clinical presentation and progress of coronavirus disease 19 (COVID-19), caused by SARS-CoV-2 and responsible for the current global pandemic, might significantly depend on the fine balance between different degrees of complement activation. For example, an excessive and unrestrained complement activation might favour the development of a systemic pro-inflammatory, pro-oxidant, and pro-coagulant state with multi-organ dysfunction and increased risk of adverse clinical outcomes (4–6). The measurement of specific components, e.g., the complement proteins C3 and C4, using immunoassays is routinely used in clinical practice to determine and monitor complement activation. The latter is reflected by a reduction in serum concentrations of C3 and/or C4 due to increased product consumption (2, 3). The assessment of C3 and C4 in COVID-19 patients might provide useful information regarding the balance between ‘physiological’ *vs.* ‘abnormal’ complement activation and overall clinical risk.

We sought to investigate the clinical role of complement activation in COVID-19 by conducting a systematic review and meta-analysis of studies reporting serum complement C3 and C4 concentrations in patients with different disease severity and survival status during follow-up. We speculated that patients with severe disease and/or reduced survival had lower complement C3 and C4 when compared to those with milder disease and favourable outcomes, reflecting a state of unrestrained complement activation in the former. Furthermore, a meta-regression analysis was performed to investigate possible associations between the effect size of the between-group differences in C3 and C4 concentrations and several clinical and demographic factors and markers of organ damage, inflammation, and coagulation.

## Materials & Methods

### Search Strategy, Eligibility Criteria & Study Selection

We conducted a systematic literature search, using the terms “complement C3” or “complement component 3” or “complement C4” or “complement component 4” and “coronavirus disease 19” or “COVID-19”, in the electronic databases PubMed, Web of Science and Scopus, from January 2020 to February 2021, to identify peer-reviewed studies reporting serum complement C3 and C4 concentrations in COVID-19 patients according to disease severity and/or survival status (PROSPERO registration number: CRD42021239634). The references of the retrieved articles were also reviewed to identify additional studies. Eligibility criteria for inclusion were a) reporting continuous data on serum C3 and C4 concentrations in COVID-19 patients, b) investigating COVID-19 patients with different degree of disease severity and/or survival status, c) adult patients, d) English language, e) ≥10 patients, and f) full-text available (7). Two investigators independently screened the abstracts. If relevant, the full-text articles were independently reviewed. A third investigator was involved in case of disagreement. Data extracted from each article included country where the study was conducted, endpoint, study design, number of participants, age, sex, serum C3 and C4 concentrations, and parameters used in meta-regression analysis (see specific details under Statistical analysis). The Newcastle-Ottawa scale was used to assess the risk of bias of each study, with a score ≥6 indicating low risk, 4-5 moderate risk, and <4 high risk (7–9). Certainty of evidence was assessed using the Grades of Recommendation, Assessment, Development and Evaluation (GRADE) Working Group system, which considers the following criteria: study design (randomized vs. observational), risk of bias (Newcastle-Ottawa scale), unexplained heterogeneity, indirectness of evidence, imprecision of results (sample size, 95% confidence interval width and threshold crossing), effect size (small, SMD <0.5, medium, SMD 0.5-0.8, and large, SMD >0.8) (10), and high probability of publication bias (11–13).

### Statistical Analysis

Standardized mean differences (SMD) with 95% confidence intervals (CIs) were calculated to build forest plots of continuous data and evaluate differences in serum C3 and C4 concentrations between COVID-19 patients with low *vs.* high severity or survivor *vs.* non-survivor status, with a p-level of significance set at < 0.05. Means and standard deviations were extrapolated from medians and interquartile ranges (14), or from graph data using the Graph Data Extractor software (7). The Q-statistic was used to test the heterogeneity of SMD across studies (p<0.10). Inconsistency across studies was evaluated through the I^2^ statistic, with I^2^<25% indicating no heterogeneity, I^2^ 25-50% moderate heterogeneity, I^2^ 50-75% large heterogeneity, and I^2^>75% extreme heterogeneity (7, 15, 16). A random-effects model was used to calculate the pooled SMD and 95% CIs in the presence of significant heterogeneity. Sensitivity analyses were performed to evaluate the influence of individual studies on the overall effect size using the leave-one-out method (7, 17). The presence of publication bias was assessed with the Begg’s adjusted rank correlation test and the Egger’s regression asymmetry test (p<0.05 for both) (18, 19). The Duval and Tweedie “trim and fill” procedure, a funnel-plot-based method of testing and adjusting for publication bias, was also used (7, 20). This is a nonparametric (rank-based) data augmentation technique that increases the observed data, so that the funnel plot is more symmetric, and recalculates the pooled SMD based on the complete data. Univariate meta-regression analysis was used to identify possible contributors to between-study variance. In particular, we investigated associations between the SMD and biologically and/or clinically plausible factors, including age, gender, clinical endpoint, diabetes, hypertension and cardiovascular disease, biomarkers of inflammation (C-reactive protein, CRP, white blood cell count, WBC, neutrophils, lymphocytes), liver damage (aspartate aminotransferase, AST, alanine aminotransferase, ALT, albumin), renal damage (serum creatinine), tissue damage (lactate dehydrogenase, LDH), and pro-thrombotic tendency (D-dimer, pro-thrombin time). Statistical analyses were performed using Stata 14 (STATA Corp., College Station, TX, USA). The study was compliant with the PRISMA 2020 statement regarding the reporting of systematic reviews and meta-analyses (21).

## Results

### Study Selection

**Figure 1:**
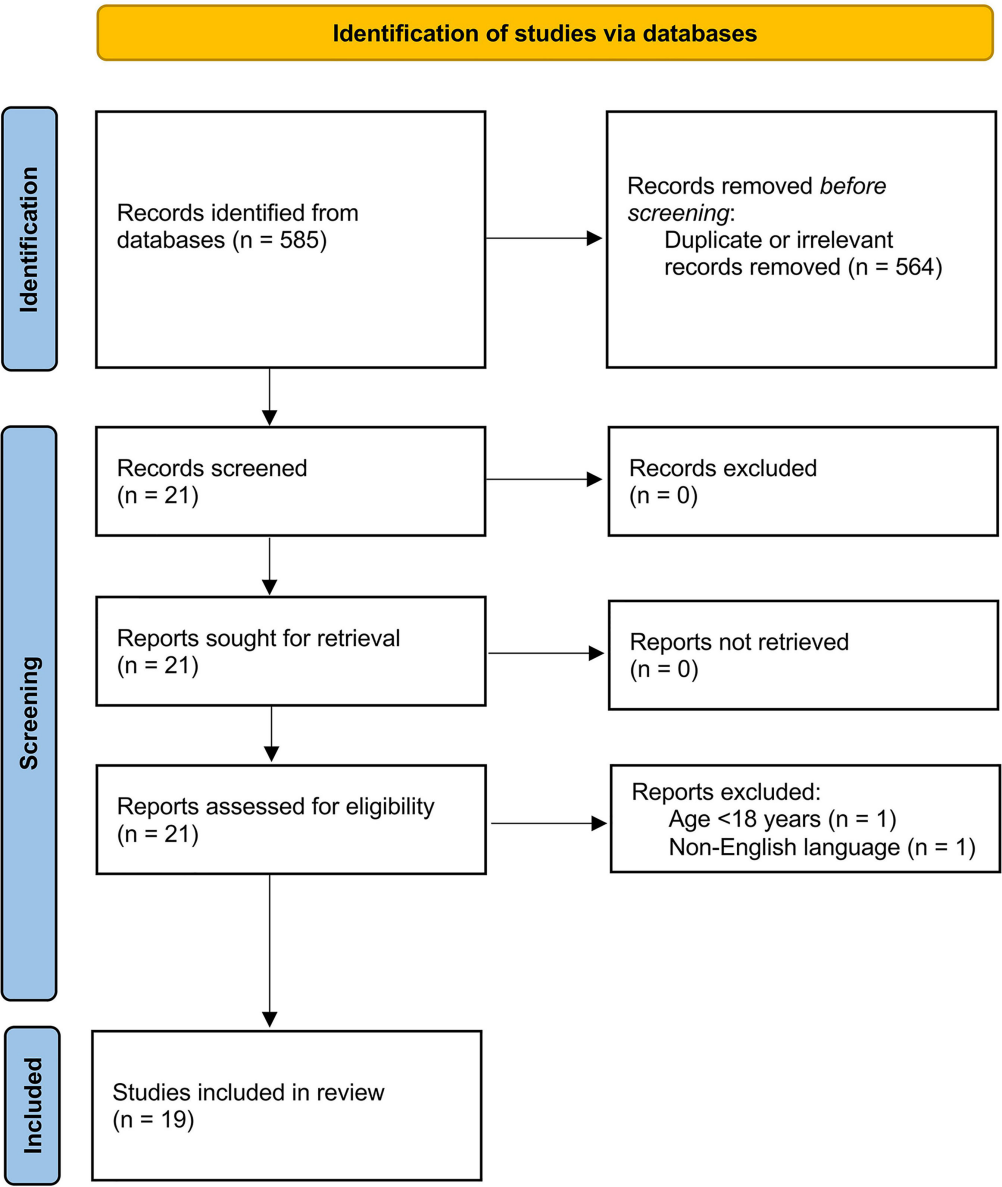
PRISMA 2020 flow diagram.

We initially identified 585 studies. A total of 564 studies were excluded because they were either duplicates or irrelevant. After a full-text review of the remaining 21 articles, two were excluded because they did not meet the inclusion criteria. Thus, 19 studies were included in the meta-analysis (**Figure 1**) (22–40). These studies enrolled 3,764 COVID-19 patients, 2,643 (48% males, mean age 54 years) with low disease severity or survivor status and 1,121 (58% males, mean age 65 years) with high severity or non-survivor status during follow up.

### Study Characteristics

One study was conducted in Turkey (23), one in Spain (33), and the remaining 17 in China (22, 24–32, 34–40). Of the 17 studies conducted in China, 10 were from the Renmin Hospital, Wuhan (24–29, 35, 38–40) (**Supplementary Table**). One study was prospective (29), 16 retrospective (22–26, 28, 30–35, 37–40), whereas the remaining two did not provide information regarding the study design (27, 36). Eleven studies assessed disease severity based on current clinical guidelines (26–29, 31–34, 37, 38, 40), one on clinical progress (36), one on ICU transfer (23), one on hospital length of stay (30), whereas the remaining six assessed survival status (**Table 1**) (22, 24, 25, 35, 39, 40). Seventeen studies reported C3 and C4 concentrations measured within the first 24-48 h from admission (22–27, 30–40), whilst the remaining two did not specify the collection time (28, 29).

**Table 1:** Characteristics of the studies included in the meta-analysis.

				Low severity or survivor					High severity or non-survivor				
First Author, Country (ref)	Endpoint	Design	NOS (stars)	n	Age (Years)	Gender (M/F)	C3 g/L (Mean ± SD)	C4 g/L (Mean ± SD)	n	Age (Years)	Gender (M/F)	C3 g/L (Mean ± SD)	C4 g/L (Mean ± SD)
Chen T et al, China (22)	Survival	R	6	161	51	88/73	0.90 ± 0.15	0.27 ± 0.07	113	68	83/30	0.77 ± 0.22	0.23 ± 0.07
Dheir H et al, Turkey (23)	ICU transfer	R	3	28	NR	NR	1.48 ± 0.36	0.31 ± 0.12	29	NR	NR	1.15 ± 0.36	0.23 ± 0.16
Fang S et al, China (24)	Survival	R	7	169	51	71/98	1.02 ± 0.29	0.26 ± 0.15	67	72	42/25	0.94 ± 0.37	0.24 ± 0.19
Fu YQ et al, China (25)	Survival	R	7	71	62	38/33	1.03 ± 0.18	0.24 ± 0.09	14	67	11/3	0.95 ± 0.17	0.25 ± 0.14
Han Y et al, China (26)	Disease severity	R	7	59	61	29/30	1.03 ± 0.23	0.26 ± 0.15	48	67	31/17	1.07 ± 0.28	0.28 ± 0.08
He B et al, China (27)	Disease severity	NR	5	32	42	15/17	1.07 ± 0.22	0.27 ± 0.07	21	57	13/8	0.87 ± 0.22	0.17 ± 0.07
He R et al, China (28)	Disease severity	R	5	135	43	42/93	0.84 ± 0.17	0.23 ± 0.10	69	61	37/32	0.90 ± 0.16	0.26 ± 0.09
Li L et al, China (29)	Disease severity	P	5	60	45	32/28	0.83 ± 0.25	0.26 ± 0.14	12	52	7/5	0.51 ± 0.12	0.10 ± 0.03
Lin P et al, China (30)	Length of stay*	R	7	20	35	8/12	0.78 ± 0.14	0.18 ± 0.05	27	41	16/11	0.89 ± 0.24	0.26 ± 0.13
Liu J et al, China (31)	Disease severity	R	5	27	43	8/19	0.80 ± 0.20	0.30 ± 0.10	23	60	7/6	0.80 ± 0.10	0.30 ± 0.10
Liu SL et al, China (32)	Disease severity	R	5	194	43	91/103	1.17 ± 0.28	0.24 ± 0.04	31	64	17/14	1.13 ± 0.24	018 ± 0.06
Marcos-Jiménezet al, Spain (33)	Disease severity	R	5	235	62	132/103	1.22 ± 0.28	0.28 ± 0.10	41	68	31/10	0.96 ± 0.32	0.19 ± 0.12
Qin C et al, China (34)	Disease severity	R	5	166	53	80/86	0.88 ± 0.17	0.26 ± 0.08	286	61	155/131	0.89 ± 0.17	0.26 ± 0.08
Qin W et al, China (35)	Survival	R	7	239	63	113/126	1.01 ± 0.21	0.25 ± 0.08	23	69	10/13	0.96 ± 0.16	0.27 ± 0.1
Xie J et al, China (36)	Disease progression	NR	6	75	51	45/30	1.33 ± 0.24	0.37 ± 0.11	29	66	18/11	1.19 ± 0.18	0.35 ± 0.08
Xie L et al, China (37)	Disease severity	R	5	322	NR	168/154	1.20 ± 0.22	0.34 ± 0.33	51	NR	29/22	1.07 ± 0.24	0.28 ± 0.09
Yuan X et al, China (38)	Disease severity	R	5	60	66	30/30	0.92 ± 0.25	0.23 ± 0.08	56	68	26/30	0.84 ± 0.12	0.21 ± 0.11
Zhao Y et al, China (39)	Survival	R	7	414	54	184/230	0.99 ± 0.21	0.25 ± 0.10	125	70	71/54	0.89 ± 0.22	0.23 ± 0.10
Zou L et al. (a), China (40)	Disease severity	R	6	69	60	34/35	1.06 ± 0.18	0.27 ± 0.10	52	70	32/20	0.94 ± 0.16	0.26 ± 0.10
Zou L et al. (b), China (40)	Survival	R	6	107	64	57/50	1.03 ± 0.18	0.27 ± 0.10	14	68	9/5	0.88 ± 0.13	0.21 ± 0.08

ICU, intensive care unit; NOS, Newcastle-Ottawa quality assessment scale for case-control studies; R, retrospective; P, prospective; NR, not reported; *, <21 vs. ≥21 days.

### Risk of Bias

The risk of bias was considered low in nine studies (22, 24–26, 30, 35, 36, 39, 40), moderate in nine (27–29, 31–34, 37, 38) and high in the remaining one (23).

### Results of Individual Studies and Syntheses

#### Complement C3

**Figure 2:**
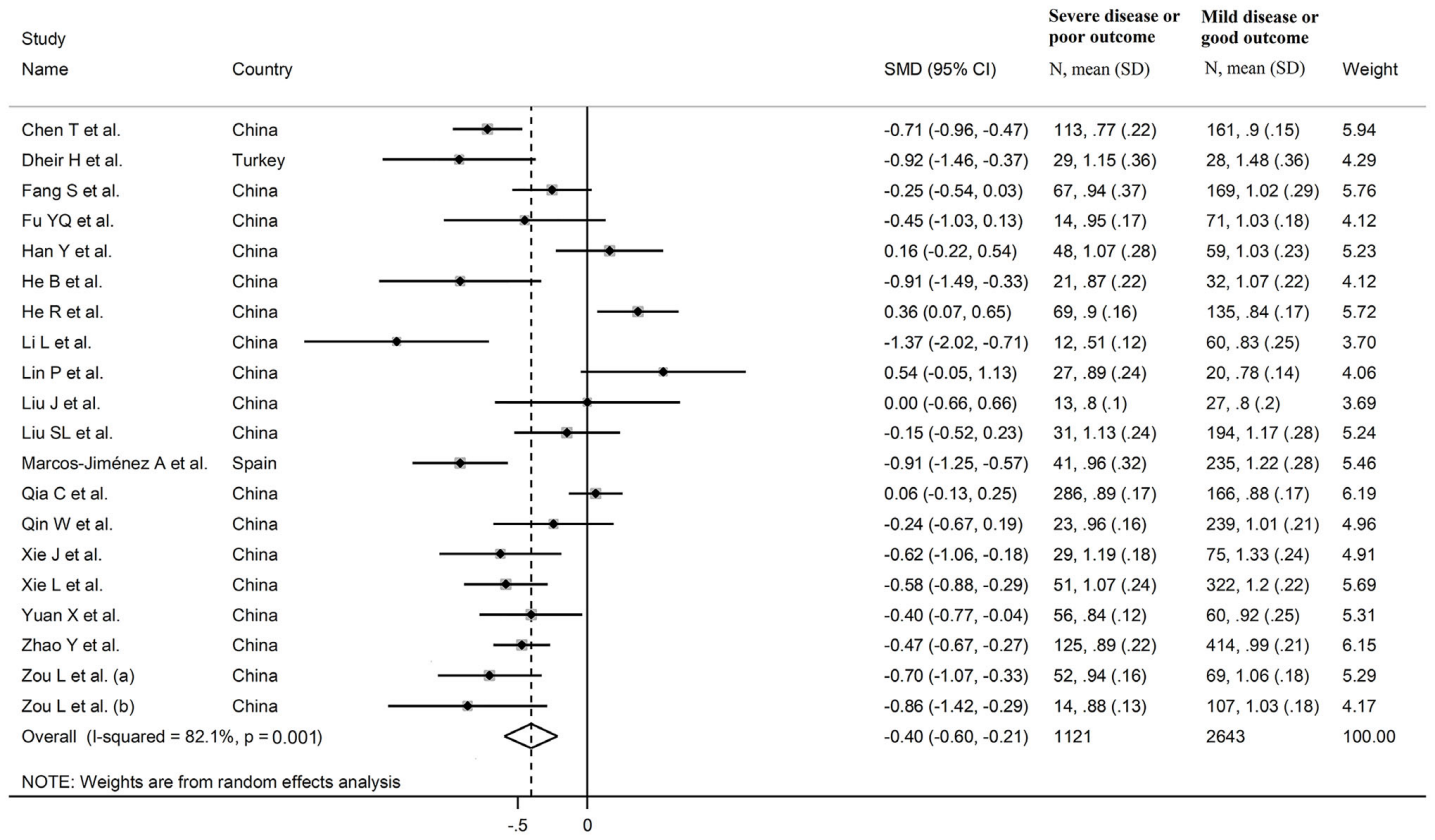
Forest plot of studies reporting complement C3 concentrations in patients with COVID-19.

The overall SMD in complement C3 concentrations between COVID-19 patients with low *vs.* high severity or survivor vs. non-survivor status in the 19 studies is shown in **Figure 2**. In 15 studies, patients with high severity or non-survivor status had lower C3 concentrations when compared to those with low severity or survivor status (mean difference range, -0.37 to -0.15) (22–25, 27, 29, 32, 33, 35–40), with a significant difference in 11 (22, 23, 27, 29, 32, 33, 35–40). No between-group difference was reported in one study (mean difference 0.00) (31). By contrast, in the remaining four studies, the C3 concentration was lower in patients with low severity or survivor status (mean difference range, 0.06 to 0.54) (26, 28, 30, 33), although only one study reported a significant difference (28). The pooled results confirmed that C3 concentrations were significantly lower in patients with high disease severity or non-survivor status during follow up (SMD -0.40, 95% CI -0.60 to -0.21, p<0.001) (**Figure 2**). Extreme heterogeneity between studies was observed (I^2^ = 82.1%, p<0.001).

**Figure 3:**
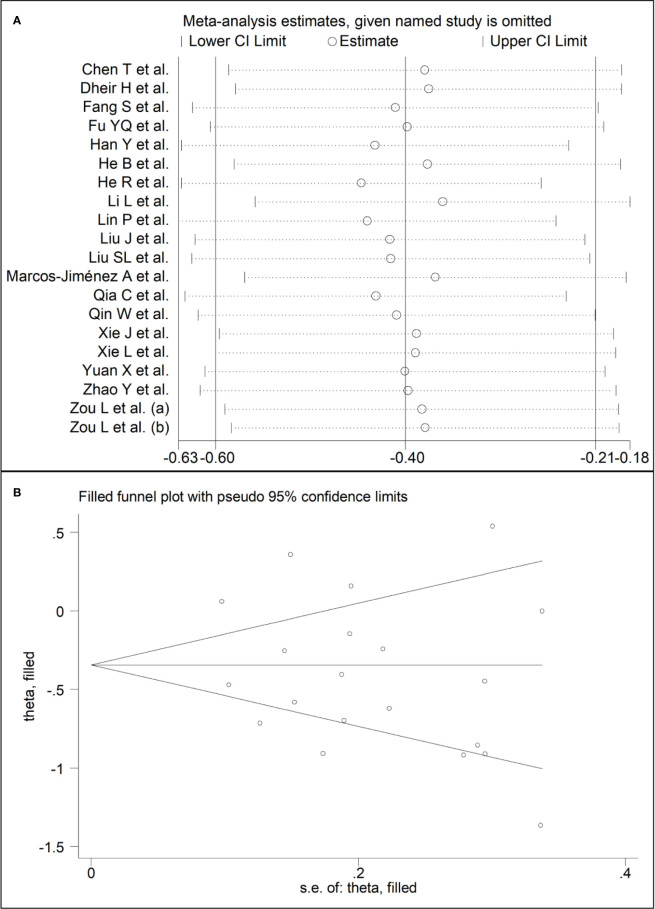
**(A)** Sensitivity analysis of the association between complement C3 and COVID-19. The influence of individual studies on the overall standardized mean difference (SMD) is shown. The middle vertical axis indicates the overall SMD, and the two vertical axes indicate the 95% confidence intervals (CIs). The hollow circles represent the pooled SMD when the remaining study is omitted from the meta-analysis. The two ends of each broken line represent the 95% CI. **(B)** Funnel plot of studies investigating low *vs.* high severity or survivor *vs.* non-survivor status after trimming and filling. Dummy studies and genuine studies are represented by enclosed circles and free circles, respectively.

**Figure 4:**
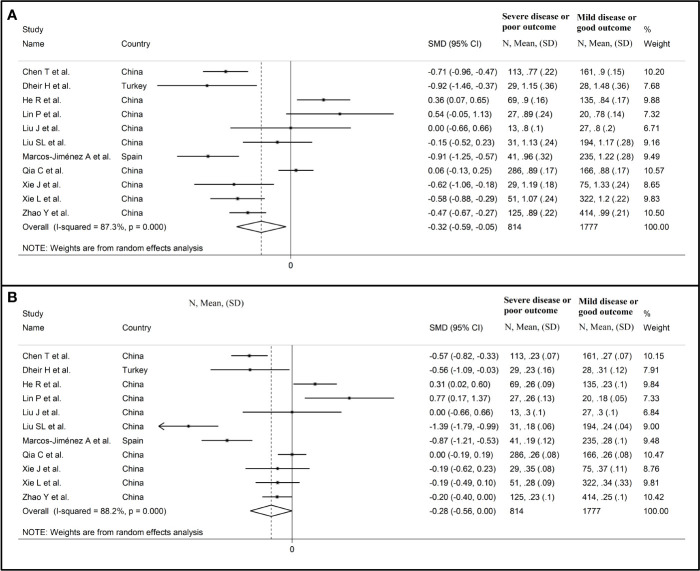
Forest plot of studies reporting complement C3 **(A)** and C4 concentrations **(B)** after removing those conducted at the Renmin Hospital, Wuhan, barring the largest ones for disease severity (28) and survival status (39).

Sensitivity analysis, performed by sequentially removing each study and re-assessing the pooled estimates, showed that the magnitude and direction of the effect size were not substantially modified (effect size range, between -0.45 and -0.37) (**Figure 3A**) (7). Furthermore, after removing the studies conducted at the Renmin Hospital, Wuhan, barring the largest ones for disease severity (28) and survival status (39), the SMD remained significant (SMD -0.32, 95% CI -0.59 to -0.05, p=0.02; I^2^ = 87.3%, p<0.001) (**Figure 4A**).

Complement C3 concentrations remained significantly lower (SMD -0.42, 95% CI -0.65 to -0.20, p<0.001; I^2^ = 80.9%, p<0.001) in patients with high severity or non-survivor status after removing three relatively large studies that accounted for nearly 36% of the total sample size (34, 37, 39).

In univariate meta-regression, the SMD was significantly associated with WBC (t=-2.39, p=0.03), CRP (t=3.08, p=0.008), and pro-thrombin time (t=3.95, p=0.004), with a further trend observed with neutrophils (t=-2.13, p=0.052). By contrast, no significant correlations were observed with age (t=0.78, p= 0.45), gender, (t=1.75, p=0.10), lymphocytes (t=0.60, p=0.56), AST (t=0.04, p=0.97), ALT (t=1.19, p=0.26), LDH (t=-0.14, p=0.89), D-dimer (t=-1.32, p=0.17), albumin (t=1.08, p=0.31), creatinine (t=0.31, p=0.76), diabetes (t=-0.23, p=0.82), hypertension (t=1.23, p=0.24) and cardiovascular disease (t=0.29, p=0.78).

**Figure 5:**
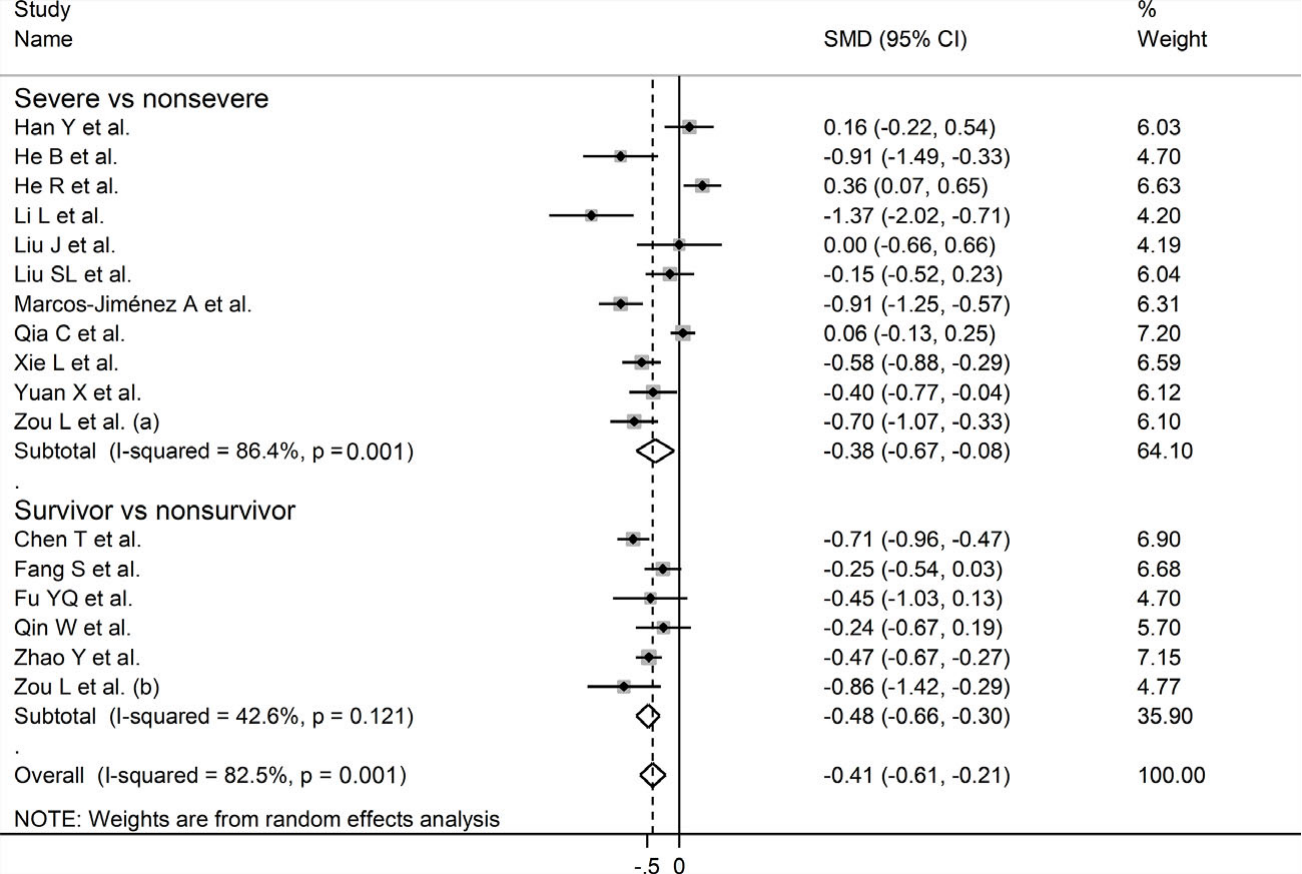
Forest plot of studies reporting complement C3 concentrations in patients with COVID-19 according to disease severity or survival status. The diamond represents the point estimate and confidence intervals after combining and averaging the individual studies. The vertical line through the vertical points of the diamond represents the point estimate of the averaged studies.

In sub-group analysis, the pooled SMD in studies investigating disease severity (SMD -0.38, 95% CI -0.67 to -0.08, p<0.001; I^2^ = 86.4, p=0.013) was non-significantly higher than that in studies investigating survival status (SMD -0.48, 95% CI -0.61 to -0.21, p<0.001; I^2^ = 42.6, p=0.12; t=-0.54, p=0.60) (**Figure 5**). However, the between-study variance was substantially lower in studies investigating survival status (I^2^ = 42.6 *vs.* I^2^ = 86.4).

#### Complement C4

**Figure 6:**
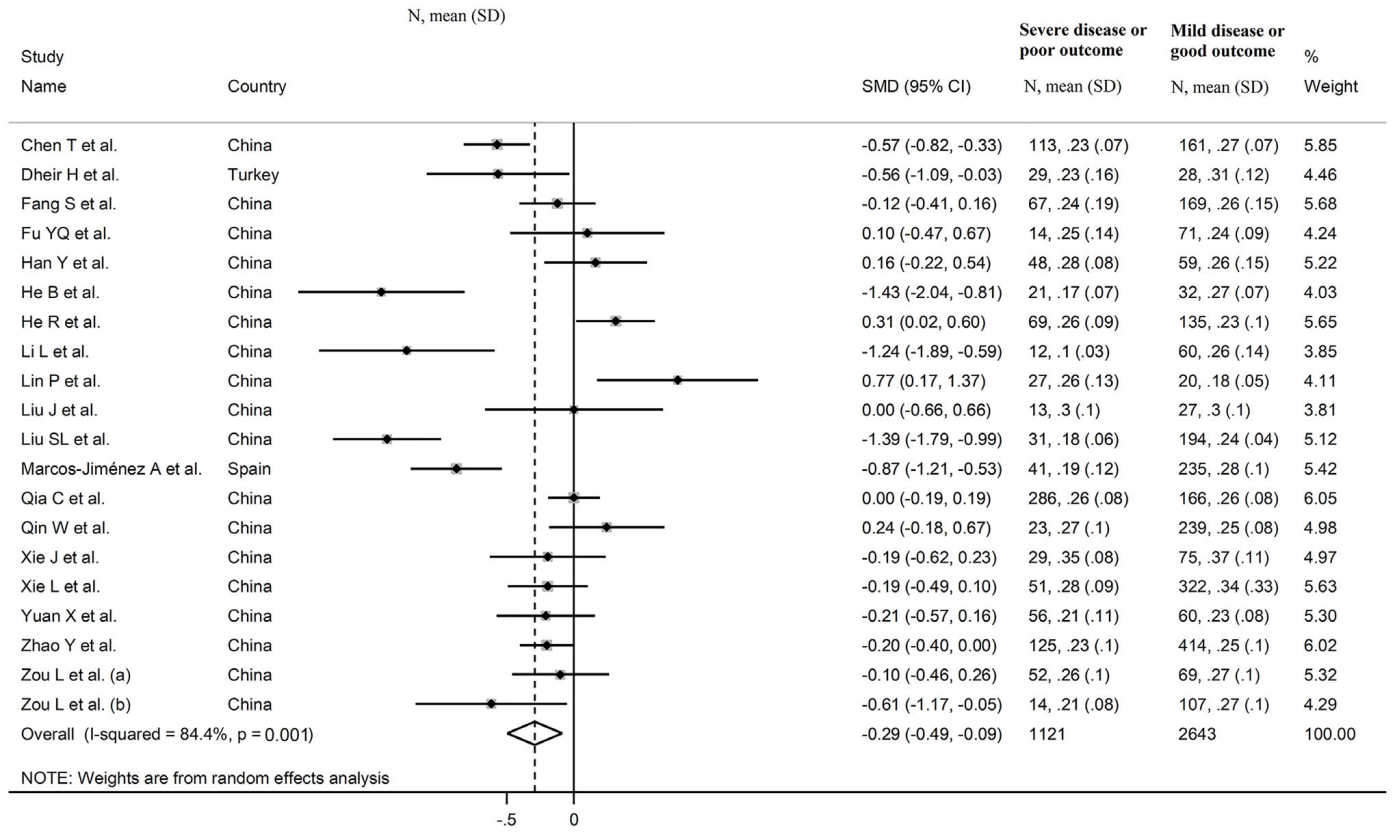
Forest plot of studies reporting complement C4 concentrations in patients with COVID-19.

The overall SMD in complement C4 concentrations between COVID-19 patients with low vs. high severity or survivor vs. non-survivor status in the 19 studies is shown in **Figure 6**. In 13 studies, patients with high severity or non-survivor status had lower C4 concentrations when compared to those with low severity or survivor status (mean difference range, -1.43 to -0.10) (22–24, 27, 29, 32, 33, 36–40), with a significant difference in seven (22, 23, 27, 29, 32, 33, 40). No between-group difference was observed in two studies (mean difference 0.00) (31, 34), whereas in the remaining five the C4 concentration was lower in patients with low severity or survivor status (mean difference range, 0.10 to 0.77) (25, 26, 28, 30, 35), with a significant difference in two (28, 30). The pooled results confirmed that C4 concentrations were significantly lower in patients with high severity or non-survivor status during follow up (SMD -0.29, 95% CI -0.49 to -0.09, p=0.005) (**Figure 6**). Extreme heterogeneity between studies was observed (I^2^ = 84.4%, p<0.001).

**Figure 7:**
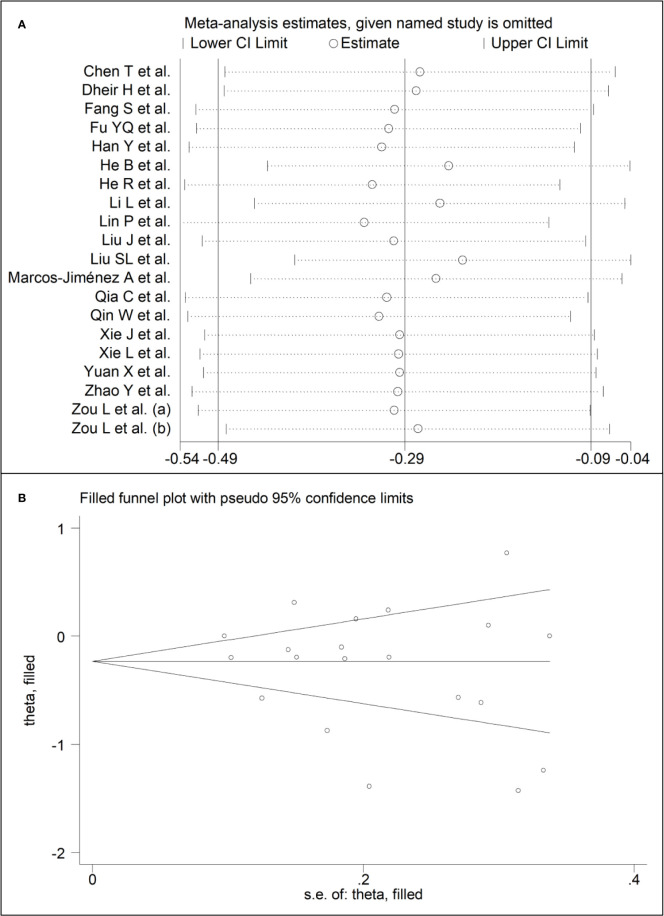
**(A)** Sensitivity analysis of the association between complement C4 and COVID-19. The influence of individual studies on the overall standardized mean difference (SMD) is shown. The middle vertical axis indicates the overall SMD, and the two vertical axes indicate the 95% confidence intervals (CIs). The hollow circles represent the pooled SMD when the remaining study is omitted from the meta-analysis. The two ends of each broken line represent the 95% CI. **(B)** Funnel plot of studies investigating low *vs.* high severity or survivor *vs.* non-survivor status after trimming and filling. Dummy studies and genuine studies are represented by enclosed circles and free circles, respectively.

Sensitivity analysis, performed by sequentially removing each study and re-assessing the pooled estimates, showed that the magnitude and direction of the effect size was not substantially altered (effect size range, between -0.31 and -0.23) (**Figures 7A, B**) (7). Furthermore, after removing the studies conducted at the Renmin Hospital, Wuhan, barring the largest ones for disease severity (28) and survival status (39), the SMD remained substantially unchanged, albeit borderline significant (SMD -0.28, 95% CI -0.56 to 0.00, p=0.05; I^2^ = 88.2%, p<0.001) (**Figure 4B**).

Similar to C3, complement C4 concentrations remained significantly lower (SMD -0.33, 95% CI -0.59 to -0.06, p=0.015; I^2^ = 85.9%, p<0.001) in patients with high severity or non-survivor status after removing three relatively large studies accounting for nearly 36% of the total sample size (34, 37, 39).

In meta-regression, CRP (t=2.58, p=0.02), pro-thrombin time (t=-2.53, p=0.03), D-dimer (t=-2.78, p=0.02), and albumin (t=3.66, p=0.006) were significantly associated with the pooled SMD. A trend toward a significant association was also observed between effect size and WBC (t=-1.93, p=0.07) and neutrophils (t=-2.07, p=0.06). By contrast, no significant correlations were observed between the SMD and age (t=-1.00, p=0.33), gender, (t=1.05, p=0.31), lymphocytes (t=0.72, p=0.49), AST (t=-0.16, p=0.87), ALT (t=-0.94, p=0.36), LDH (t=-0.08, p=0.93), creatinine (t=0.40, p=0.70), diabetes (t=-0.55, p=0.54), hypertension (t=0.61, p=0.55) and cardiovascular disease (t=-0.21, p=0.84).

**Figure 8:**
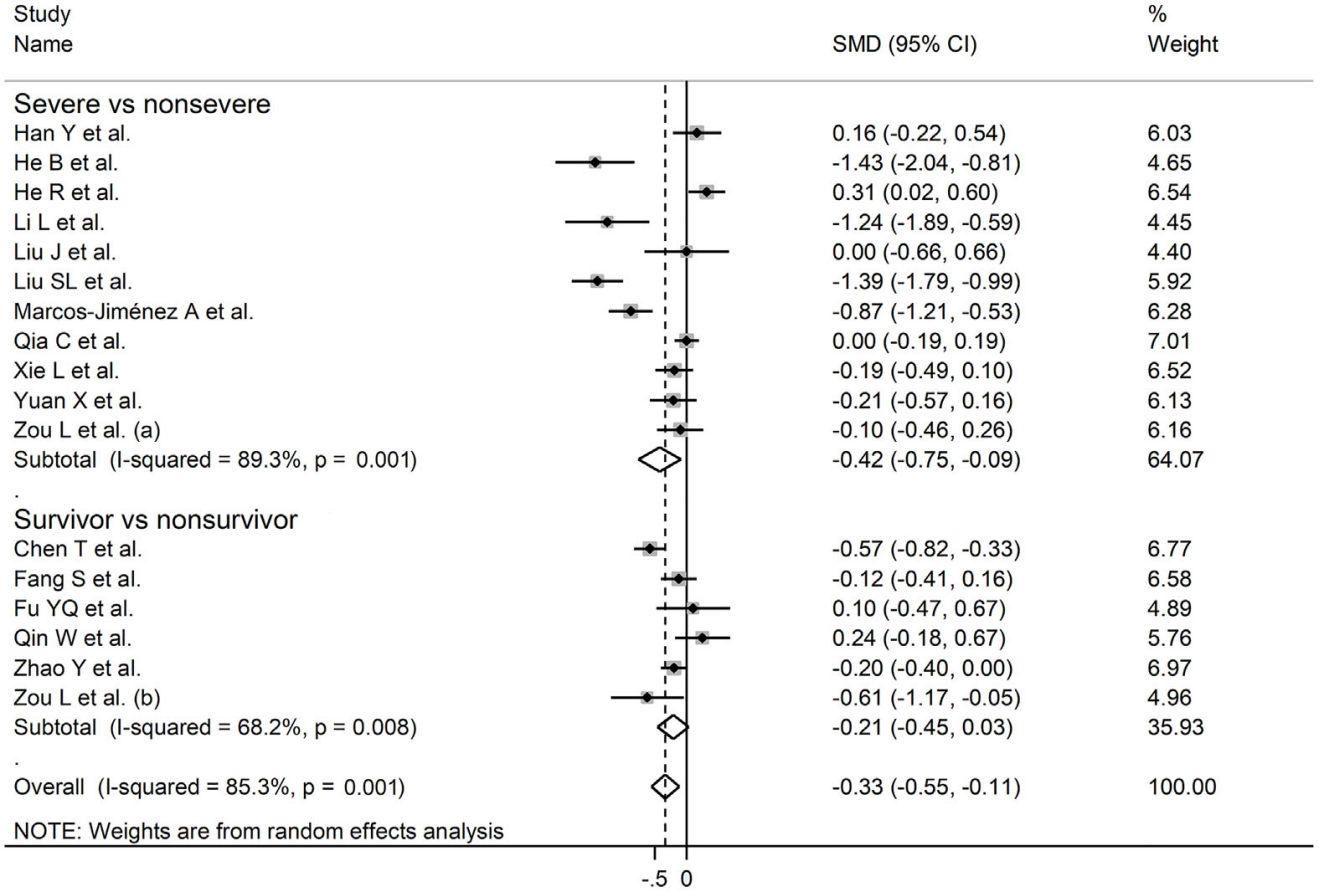
Forest plot of studies reporting complement C4 concentrations in patients with COVID-19 according to disease severity or survival status. The diamond represents the point estimate and confidence intervals after combining and averaging the individual. The vertical line through the vertical points of the diamond represents the point estimate of the averaged studies.

In sub-group analysis, the pooled SMD in studies reporting disease severity (SMD -0.42, 95% CI -0.75 to -0.09, p<0.001; I^2^ = 89.3, p=0.013) was non-significantly lower than that in studies reporting survival status (SMD -0.21, 95% CI -0.45 to 0.03, p=0.09; I^2^ = 68.2, p=0.008; t=0.78, p=0.44) (**Figure 8**). However, the between-study variance was relatively lower in studies reporting survival (I^2^ = 68.2 *vs.* I^2^ = 89.3).

### Publication Bias

#### Complement C3

There was no publication bias according to the Begg’s (p=0.63) and Egger’s (p=0.30) t-tests. Accordingly, the trim‐and‐fill analysis showed that no study was missing or should be added (**Figure 3B**) (7).

#### Complement C4

The Begg’s (p=0.35) and Egger’s (p=0.37) t-tests did not show publication bias. Accordingly, the trim‐and‐fill analysis showed that no study was missing or should be added (**Figure 7B**) (7).

### Certainty of Evidence

The initial level of certainty for serum C3 and C4 SMD values was considered low because of the observational nature of the selected studies (rating 2, ⊕⊕⊝⊝). After considering the presence of a low-moderate risk of bias in 18 out of 19 studies (no rating change required), a generally extreme and unexplained heterogeneity (serious limitation, downgrade one level), lack of indirectness (no rating change required), the relatively low imprecision (relatively narrow confidence intervals without threshold crossing, upgrade one level), the relatively small effect size (SMD between -0.29, C4, and -0.40, C3, no rating change required) (10), and absence of publication bias (upgrade one level), the overall level of certainty was considered moderate (rating 3, ⊕⊕⊕⊝).

## Discussion

In this systematic review and meta-analysis, we observed that the serum concentrations of complement C3 and C4 were significantly lower in COVID-19 patients with more severe disease or who died during follow up when compared to those with milder disease or survivor status. The magnitude of the observed SMD values, -0.40 for C3 and -0.29 for C4, suggests that the between-group differences are significant either from a biological and a clinical point of view (10). The between-group heterogeneity was extreme however the sequential omission of individual studies did not exert tangible effects on the overall SMD value. Furthermore, there was no evidence of publication bias. Meta-regression analysis showed significant associations between the SMD of C3 and white blood cell count, CRP, and pro-thrombin time, and between the SMD of C4 and CRP, pro-thrombin time, D-dimer, and albumin.

The measurement of the serum concentrations of complement C3 and C4 is useful in the diagnosis and the monitoring of blood associated infectious diseases and immune complex diseases. By and large, C3 is often decreased through consumption during infections whereas a combined reduction in C3 and C4 is observed in immune complex disease (1, 2). The assessment the complement system during SARS-CoV-2 has gained considerable attention because of the potential adverse consequences of an unrestrained activation of the system on the structural and functional integrity of different organs and tissues. This proposition is supported by the results of studies reporting a beneficial effect of corticosteroid treatment in patients with COVID-19, which suggests that the organ and tissue injury is not directly caused by viral infection but, rather, is the consequence of an excessive host immune response. This, also reflected by the activation of the complement system, facilitates the release of pro-inflammatory cytokines and, consequently, a state of intra-vascular coagulation and cell death (5, 41). Autoptic studies in patients with COVID-19 have shown the accumulation of complement components in the lungs and kidneys, and concomitant evidence of tissue injury in these organs, confirming the detrimental role of excessive complement activation in this group (42, 43). The latter is likely to involve the contribution of C3b, a product of the C3 convertases, to the formation of complement C5 convertases. These, in turn, cleave C5 into C5a, an anaphylatoxin that exacerbates the activity of pro-inflammatory pathways, and C5b, which triggers the downstream events of complement activation, i.e., the formation of the membrane-attack complex and, by forming C5b-9, the induction of cell injury that also involves the endothelium (6, 44). The observed associations, in meta-regression analysis, between the SMD values of C3 and C4 and CRP, pro-thrombin time, and D-dimer (C4 only) further support the presence of a complex, yet relevant from a pathophysiologic point of view, interplay between the activation of the complement system, inflammatory and pro-coagulant pathways, on one hand, and the degree of disease severity and its clinical consequences, on the other, in patients with COVID-19.

The extreme between-study heterogeneity observed in our analyses represents a significant limitation that reduces to a certain extent the generalizability of the results. It is possible that other, unreported factors might have contributed to the observed heterogeneity. At the same time, there was no evidence of publication bias and the overall effect size was not affected in sensitivity analysis. Another significant limitation is that 3,501 of the 3,764 patients were Chinese, and 10 studies were conducted in the same hospital (Renmin Hospital, Wuhan). While similar SMD values were observed after removing the studies from this hospital, barring the largest ones for disease severity (28) and survival status (39), the possibility of duplicate data cannot be completely ruled out. Furthermore, no selected study performed a serial measurement of complement component concentrations during hospitalization. This might provide additional information regarding possible clinical deterioration. In one study investigating serial concentrations of C3a and C5a, significant elevations of the latter, but not of the former, preceded the onset of clinical deterioration (45). Further studies are required to determine whether serial measurements of complement components, including C3 and C4, provide additional prognostic information to that of single measurements on admission.

The increasing evidence of an unrestrained complement activation in severe COVID-19 has also prompted the search for targeted therapies that suppress this phenomenon. Inhibitors targeting the early steps of complement activation have shown, in small studies, promising effects on inflammatory markers, respiratory function, and clinical outcomes (46). Larger, randomized controlled studies, are urgently required to explore the full potential of this treatment strategy in patients with different degrees of COVID-19 severity and complement activation.

In conclusion, our systematic review and meta-analysis with meta-regression has shown that lower serum concentrations of C3 and C4, indicating excessive complement activation and product consumption, are significantly associated with the presence of severe disease and increased mortality in patients with COVID-19. Additional studies are required to determine whether single or serial measurement of complement components, with or without other clinical, demographic, and biochemical characteristics, can further increase our capacity to predict COVID-19 severity and adverse clinical outcomes.

## Data Availability Statement

The original contributions presented in the study are included in the article/**Supplementary Material**. Further inquiries can be directed to the corresponding author.

## Author Contributions

Initial idea: AZ and AM. Data collection and analysis: AZ. Data interpretation: AZ and AM. Writing - first draft: AM. Writing - Review & Editing: AZ and AM. All authors contributed to the article and approved the submitted version.

## Conflict of Interest

The authors declare that the research was conducted in the absence of any commercial or financial relationships that could be construed as a potential conflict of interest.
