# Supplementary material for: Serum Complement C3 and C4 and COVID-19 Severity and Mortality: A Systematic Review and Meta-Analysis With Meta-Regression
Source: Front Immunol. 2021 Jun 7;12:696085. doi: 10.3389/fimmu.2021.696085 (PMC8215447; doi:10.3389/fimmu.2021.696085)
Supplement: Supplementary file 1 [file Table_1.docx]

**Supplementary Table.** Characteristics of studies conducted in China*****

| **Authors** | **Hospital/University and Department** | **Period of enrolment** |
| --- | --- | --- |
| Chen T et al. | Department and Institute of Infectious Disease, Tongji Hospital, Tongji Medical, Wuhan  Department of Respiratory Disease, Tongji Hospital, Wuhan  Department of Emergency Medicine, Tongji Hospital, Wuhan  Department of Paediatrics, Tongji Hospital, Wuhan | January 13 - February 12, 2020 |
| Fang S et al. | **Department of Pain, Renmin Hospital of Wuhan University, Wuhan**  Department of Gastroenterology, Zhongnan Hospital of Wuhan University, Wuhan  Department of Critical Care Medicine, University-Town Hospital of Chongqing Medical University, Chongqing  **Department of Anesthesiology, Renmin Hospital of Wuhan University, Wuhan** | January 31 - February 20, 2020 |
| Fu YQ et al. | Department of Critical Care Medicine, Children’s Hospital, Chongqing Medical University, Chongqing  Ministry of Education Key Laboratory of Child Development and Disorders, Chongqing  National Clinical Research Center for Child Health and Disorders (Chongqing), Chongqing  **Department of Ophthalmology, Renmin Hospital of Wuhan University, Wuhan** | February 4 - February 16, 2020 |
| Han Y et al. | Emergency Department, Zhongshan Hospital, Fudan University, Shanghai  **Department of Radiology, Renmin Hospital of Wuhan University, Wuhan** | February 1 - March 1, 2020 |
| He B et al. | **Department of Pediatrics, Renmin Hospital of Wuhan University, Wuhan**  Department of Biology and Genetics, Wuhan University of Science and Technology, Wuhan  Institute of Biology and Medicine, Wuhan University of Science and Technology, Wuhan  Department of Medicine, Hubei Maternal and Child Health Hospital, Wuhan | Patients hospitalized after February 1, 2020 |
| He R et al. | **Department of Thoracic Surgery, Renmin Hospital of Wuhan University, Wuhan** | January 10 - February 13, 2020 |
| Li L et al. | **Department of Ophthalmology, Renmin Hospital of Wuhan University, Wuhan** | January 27 - March 28, 2020 |
| Lin P et al. | Department of Basic Medicine, Quanzhou Medical College, Quanzhou  Department of Infectious Diseases, The First Hospital of Quanzhou Affiliated to Fujian Medical University, Quanzhou  Department of Respiratory Diseases, The First Hospital of Quanzhou Affiliated to Fujian Medical University, Quanzhou  Department of Respiratory Diseases, Shishi City General Hospital, Quanzhou  Department of Respiratory Diseases, Anxi County Hospital, Quanzhou  Department of Clinical Laboratory, The First Hospital of Quanzhou Affiliated to Fujian Medical University, Quanzhou | January 21 - March 6, 2020 |
| Liu J et al. | Department of Infectious Diseases, Union Hospital, Tongji Medical College, Huazhong University of Science and Technology, Wuhan  Joint International Laboratory of Infection and Immunity, Huazhong University of Science and Technology, Wuhan  Department of Clinical Laboratory, Union Hospital, Tongji Medical College, Huazhong University of Science and Technology, Wuhan  Department of Hematology, Union Hospital, Tongji Medical College, Huazhong University of Science and Technology, Wuhan | January 5 - January 24, 2020 |
| Liu SL et al. | Department of Clinical Laboratory, The First Hospital of Changsha City, Hunan  Department of Clinical Laboratory, Affiliated Liutie Central Hospital of Guangxi Medical University, Liuzhou  Hunan Key Laboratory of Oncotarget Gene and Clinical Laboratory, Hunan Cancer Hospital and  the Affiliated Cancer Hospital of Xiangya School of Medicine, Central South University, Hunan  School of Chemical Engineering, China University of Mining and Technology, Jiangsu  Department of Clinical Laboratory, Affiliated Hospital of China University of Mining and Technology, Jiangsu | February 9 - March 7, 2020 |
| Qin C et al. | Department of Neurology, Tongji Hospital, Tongji Medical College, Huazhong University of Science and Technology, Wuhan  Department of Radiology, Tongji Hospital, Tongji Medical College, Huazhong University of Science and Technology, Wuhan  Department of Respiratory and Critical Care Medicine, Tongji Hospital, Tongji Medical College, Huazhong University of Science and Technology, Wuhan  Department of Emergency Medicine, Tongji Hospital, Tongji Medical College, Huazhong University of Science and Technology, Wuhan  Department of Infectious Diseases, Tongji Hospital, Tongji Medical College, Huazhong University of Science and Technology, Wuhan | January 10 - February 12, 2020 |
| Qin W et al. | Department of Critical Care Medicine, Qilu Hospital, Cheeloo College of Medicine, Shandong University, Jinan  Department of Traditional Chinese Medicine, Qilu Hospital, Cheeloo College of Medicine, Shandong University, Jinan  State Key laboratory of Biobased Material and Green Papermaking, Key laboratory of Pulp and Paper Science and Technology of Shandong Province/Ministry of education, Qilu university of Technology, Shandong Academy of Sciences  **Department of Infectious Disease, Renmin Hospital of Wuhan University, Wuhan**  The Key Laboratory of Cardiovascular Remodeling and Function Research, Chinese Ministry of Education, Chinese National Health Commission and Chinese Academy of Medical Sciences, The State and Shandong Province Joint Key Laboratory of Translational Cardiovascular Medicine, Department of Cardiology, Qilu Hospital, Cheeloo College of Medicine, Shandong University, Jinan | January 31 - March 6, 2020 |
| Xie J et al. | State Key Laboratory for Diagnosis and Treatment of Infectious Diseases, National Clinical Research Center for Infectious Diseases, Collaborative Innovation Center for Diagnosis and Treatment of Infectious Diseases, The First Affiliated Hospital, College of Medicine, Zhejiang University, Hangzhou  State Key Laboratory of Genetic Engineering, Institute of Biostatistics, School of Life Sciences, Fudan University, Shanghai  Division of Hepatobiliary and Pancreatic Surgery, Department of Surgery, Key Lab of Combined Multi-organ Transplantation of the Ministry of Health, The First Affiliated Hospital, College of Medicine, Zhejiang University, Hangzhou  Division of Endocrinology and Metabolism, Department of Internal Medicine System, The First Affiliated Hospital, College of Medicine, Zhejiang University, Hangzhou | January 10 - February 26, 2020 |
| Xie L et al. | Department of Pulmonary Medicine, Zhongshan Hospital, Fudan University, Shanghai  Department of Infectious Diseases, Shanghai Public Health Clinical Center, Shanghai  Department of Respiratory Medicine, Affiliated Hospital of Putian University, Putian  The School of Clinical Medicine, Fujian Medical University, Fuzhou, Fujian Province | January 20 - February 29, 2020 |
| Yuan X et al. | Fujian Insitute of Hematology, Fujian Medical University Union Hospital, Fuzhou  Department of Critical Care Medicine, Fujian Medical University Union Hospital, Fuzhou | February 15 - March 30, 2020  **Recruitment at Renmin Hospital of Wuhan University** |
| Zhao Y et al. | **Department of Respiratory Medicine, Renmin Hospital of Wuhan University, Wuhan** | January 13 - March 4, 2020 |
| Zou L et al. | **Department of Neurology, Renmin Hospital of Wuhan University, Wuhan**  School of Nursing, Tongji Medical College, Huazhong University of Science and Technology, Wuhan | January 16 - March 3, 2020 |

Legend: *, studies in bold characters were conducted at the Renmin Hospital, Wuhan.
